# Green Tea and Stomach Cancer -- A Short Review of Prospective Studies

**DOI:** 10.2188/jea.15.S109

**Published:** 2005-08-18

**Authors:** Yoshiharu Hoshiyama, Takeshi Kawaguchi, Yoshihiko Miura, Tetsuya Mizoue, Noritaka Tokui, Hiroshi Yatsuya, Kiyomi Sakata, Takaaki Kondo, Shogo Kikuchi, Hideaki Toyoshima, Norihiko Hayakawa, Akiko Tamakoshi, Takesumi Yoshimura

**Affiliations:** 1 Department of Public Health, Showa University School of Medicine.; 2 Department of Nursing, Saitama University Saitama.; 3 Department of Preventive Medicine, Kyushu University Graduate School of Medical Sciences.; 4 Department of Public Health/ Health Information Dynamics, Field of Social Life Science, Program in Health and Community Medicine, Nagoya University Graduate School of Medicine.; 5 Department of Public Health, Wakayama Medical University.; 6 Department of Medical Technology, Nagoya University School of Health Sciences.; 7 Department of Public Health, Aichi Medical University.; 8 Department of Epidemiology, Research Institute for Radiation Biology and Medicine, Hiroshima University.; 9 Department of Preventive Medicine/ Biostatistics and Medical Decision Making, Nagoya University Graduate School of Medicine.; 10 Fukuoka Institute of Health and Environmental Sciences.

**Keywords:** Tea, Stomach Neoplasms, the JACC study, Case-Control Studies, Prospective Studies

## Abstract

BACKGROUND: In Japan, green tea has been drunk for a long time. Because it can be drunk casually, many people love drinking it. If such green tea has an effect to prevent stomach cancer, it will be a very convenient way to prevent the disease.

METHODS: To examine the association between green tea consumption and the risk of stomach cancer, past epidemiologic studies including JACC Study were reviewed.

RESULTS: Among eight case-control studies, five showed risk reduction with a statistically significant difference, and two studies showed risk reduction without a statistically significant difference. The remaining study showed the opposite result. Among six prospective studies regarding stomach cancer, no study showed risk reduction with a statistically significant difference. Four of the six studies showed no relation. In terms of study design, prospective studies, which are considered to be more reliable than case-controlled studies, tend to show no risk reduction. The results of case-control studies and prospective studies present considerably different impressions.

CONCLUSIONS: Prospective studies showed no inverse association between the consumption of green tea and the risk of stomach cancer.

In Japan, green tea has been drunk for a long time. Because it can be drunk casually, many people love drinking it. If such green tea has an effect to prevent stomach cancer, it will be a convenient way to prevent the disease. It began to be said that green tea is effective in preventing cancer about over ten years ago. In 1988, Kono et al.^1^ conducted a case-control study on stomach cancer in Kita-Kyushu, and reported that drinking 10 or more cups of green tea per day was preventive for stomach cancer. Subsequently, animal experiments showed results supporting this, and additional studies were conducted. Some of these studies reported that green tea was preventive, but others found it was not. In 2001, Tsubono et al.^2^ reported a prospective study, conducted in Miyagi prefecture, that considered development of stomach cancer as the end point, but no preventive effect of green tea was noted. Because this study was methodologically superior to the previous studies, the effect of green tea was questioned. In this study, however, five or more cups of green tea was classified as a category, and therefore the effect from ten or more cups exclusively could not be observed, which fact Kono pointed out in the New England Journal of Medicine.^3^ The next year, Hoshiyama et al.^4^ reported the first prospective study (JACC Study) that observed an effect from 10 or more cups, nationwide, in the British Journal of Cancer. It has been shown, for more than the past ten years, in many studies using cultured cells and test animals, that polyphenol contained in green tea has an antioxidant effect. As to epidemiologic study using human groups, many studies on stomach cancer have been reported.

Further, various study designs exist, and there can be said to be a correlation between the difficulty of implementing studies and the reliability of the results. The more difficult implementation is, the higher the reliability of the study is, in general.

Eight case-control studies^1^^,^^5^^-^^11^ have been conducted till now. Among them, five studies showed risk reduction with a statistically significant difference, and two studies showed risk reduction without a statistically significant difference. The remaining study showed the opposite result. These study results can be said to indicate that there is a reduction of risk of stomach cancer from green tea consumption.

Table 1 outlines the six prospective studies^2^^,^^4^^,^^12^^-^^15^ regarding stomach cancer. The exposure measurement of these studies was unclear. As for our study, the frequency of green tea consumption was initially assessed from five possible answers, i.e., every day (more than one cup per day), 3-4 cups per week, 1-2 cups per week, 1-2 cups per month and seldom. The question was: Do you drink Japanese tea (green tea)? For those who consumed tea every day, the number of cups a day was further identified. The volume of a typical cup of green tea is 100-120 mL in Japan.

**Table 1.** Epidemiologic studies on green tea and stomach cancer/prospective studies.

Tesis author	Region	No. of cases with stomach cancer		No. of non- patients	Relative risk (95% confidence interval)		Conclusion
Galanis et al. (1998)	Japanese Hawaiians		108	11,799	Non	1.0 (reference)	No inclease in risk
					1 cup/day	1.3 (0.7-2.1)	No significant defference
					2+ cups/day	1.5 (0.9-2.3)	
					Trend	P=0.10	

Nakachi et al. (2000)	Japan (Saitama)		140	8,421	less than 4 cups/day	1.0 (reference)	Risk reduction
					4 to 9 cups/day	- -	No significant defference
					10+ cups/day	0.7 (0.23-1.88)	

Tsubono et al. (2001)	Japan (Miyagi)		419	25,892	less than 1 cup/day	1.0 (reference)	No relation
					1 to 2 cups/day	1.1 (0.8-1.6)	
					3 to 4 cups/day	1.0 (0.7-1.4)	
					5+ cups/day	1.2 (0.9-1.6)	
					Trend	P=0.13	

Nagano et al. (2001)	Japan (Hiroshima/Nagasaki)		901	37,639	0 to 1 time/day	1.0 (reference)	No relation
					2 to 4 times/day	1.0 (0.82-1.2)	
					5+ times/day	0.95 (0.76-1.2)	
					Trend	P=0.56	

Hoshiyama et al. (2002)	Japan (nationwide) (JACC Study)	Men					
			240	30,130	less than 1 cup/day	1.0 (reference)	No relation
					1 to 2 cups/day	1.6 (0.9-2.9)	
					3 to 4 cups/day	1.1 (0.6-1.9)	
					5 to 9 cups/day	1.1 (0.6-1.9)	
					10+ cups/day	1.0 (0.5-2.0)	
					Trend	P=0.634	

		women					
			119	42,362	less than 1 cup/day	1.0 (reference)	No relation
					1 to 2 cups/day	1.1 (0.5-2.5)	
					3 to 4 cups/day	1.0 (0.5-2.1)	
					5 to 9 cups/day	0.8 (0.4-1.6)	
					10+ cups/day	0.7 (0.3-2.0)	
					Trend	P=0.476	

Hoshiyama et al. (2004)	Japan (nationwide) (JACC Study)		157	285	less than 1 cup/day	1.0 (reference)	No relation
					1 to 2 cups/day	1.3 (0.6-2.8)	
					3 to 4 cups/day	1.0 (0.5-1.9)	
					5 to 9 cups/day	0.8 (0.4-1.6)	
					10+ cups/day	1.2 (0.6-2.5)	
					Trend	P=0.899	

No study showed risk reduction with a statistically significant difference. Four of the six studies showed no relation. In terms of study design, prospective studies, which are considered to be more reliable than case-controlled studies, tend to show no risk reduction. As for our study (JACC Study),^4^ after adjustment for age, smoking status, history of peptic ulcer, family history of stomach cancer along with certain dietary elements, the risks associated with drinking one or two, three or four, five to nine, and 10 or more cups of green tea per day, relative to those of drinking less than one cup per day, were 1.6 (95% confidence interval [CI]: 0.9-2.9), 1.1 (95% CI: 0.6-1.9), 1.0 (95% CI: 0.5-2.0), and 1.0 (95% CI: 0.5-2.0), respectively, in men (p for trend=0.669), and 1.1 (95% CI: 0.5-2.5), 1.0 (95% CI: 0.5-2.5), 0.8 (95% CI: 0.4-1.6), and 0.8 (95% CI: 0.3-2.1), respectively, in women (p for trend = 0.488). We found no inverse association between green tea consumption and the risk of stomach cancer death. In our study, men and women were analyzed separately, and men were considered to show no relation at all. Women, however, seemed to show lower risk, as they drink more cups of green tea, though there was no statistically significant difference. The other JACC Study^15^ is the first study to analyze the effects of the consumption of green tea while controlling *Helicobacter.pylori* infection. After adjustment for age, smoking status, *H. pyroli* infection, history of peptic ulcer, family history of stomach cancer along with certain dietary elements, the risks associated with drinking one or two, three or four, five to nine, and 10 or more cups of green tea per day, relative to those of drinking less than one cup per day, were 1.1 (95% CI: 0.5-2.5), 0.9 (95% CI: 0.5-1.89), 0.8 (95% CI: 0.4-1.6), and 1.1 (95% CI: 0.5-2.3), respectively (p for trend=0.846). We found no inverse association between green tea consumption and the risk of stomach cancer.

The results of case-control studies and prospective studies present considerably different impressions. Most of the case-control studies, if studies without a significant difference are included, showed risk reduction, while on the contrary, no prospective study indicated risk reduction with a statistically significant difference. Such conflicting results are often seen in cancer epidemiologic studies. In case-control studies, there may be a problem with the reliability of information, because information that exposes the past history is collected after cancer is diagnosed. Patients may avoid drinking green tea due to a symptom in the stomach or for some other reason, thereby seeming to show that drinking green tea has a preventive effect.

In summary, prospective studies showed no inverse association between the consumption of green tea and the risk of stomach cancer. It is hoped that studies with further higher accuracy will be conducted in the future.

## MEMBER LIST OF THE JACC STUDY GROUP

The present investigators involved, with the co-authorship of this paper, in the JACC Study and their affiliations are as follows: Dr. Akiko Tamakoshi (present chairman of the study group), Nagoya University Graduate School of Medicine; Dr. Mitsuru Mori, Sapporo Medical University School of Medicine; Dr. Yutaka Motohashi, Akita University School of Medicine; Dr. Ichiro Tsuji, Tohoku University Graduate School of Medicine; Dr. Yosikazu Nakamura, Jichi Medical School; Dr. Hiroyasu Iso, Institute of Community Medicine, University of Tsukuba; Dr. Haruo Mikami, Chiba Cancer Center; Dr. Yutaka Inaba, Juntendo University School of Medicine; Dr. Yoshiharu Hoshiyama, University of Human Arts and Sciences; Dr. Hiroshi Suzuki, Niigata University School of Medicine; Dr. Hiroyuki Shimizu, Gifu University School of Medicine; Dr. Hideaki Toyoshima, Nagoya University Graduate School of Medicine; Dr. Kenji Wakai, Aichi Cancer Center Research Institute; Dr. Shinkan Tokudome, Nagoya City University Graduate School of Medical Sciences; Dr. Yoshinori Ito, Fujita Health University School of Health Sciences; Dr. Shuji Hashimoto, Fujita Health University School of Medicine; Dr. Shogo Kikuchi, Aichi Medical University School of Medicine; Dr. Akio Koizumi, Graduate School of Medicine and Faculty of Medicine, Kyoto University; Dr. Takashi Kawamura, Kyoto University Center for Student Health; Dr. Yoshiyuki Watanabe, Kyoto Prefectural University of Medicine Graduate School of Medical Science; Dr. Tsuneharu Miki, Graduate School of Medical Science, Kyoto Prefectural University of Medicine; Dr. Chigusa Date, Faculty of Human Environmental Sciences, Mukogawa Women’s University ; Dr. Kiyomi Sakata, Wakayama Medical University; Dr. Takayuki Nose, Tottori University Faculty of Medicine; Dr. Norihiko Hayakawa, Research Institute for Radiation Biology and Medicine, Hiroshima University; Dr. Takesumi Yoshimura, Fukuoka Institute of Health and Environmental Sciences; Dr. Akira Shibata, Kurume University School of Medicine; Dr. Naoyuki Okamoto, Kanagawa Cancer Center; Dr. Hideo Shio, Moriyama Municipal Hospital; Dr. Yoshiyuki Ohno, Asahi Rosai Hospital; Dr. Tomoyuki Kitagawa, Cancer Institute of the Japanese Foundation for Cancer Research; Dr. Toshio Kuroki, Gifu University; and Dr. Kazuo Tajima, Aichi Cancer Center Research Institute.
